# Prediction model for hyperprogressive disease in non‐small cell lung cancer treated with immune checkpoint inhibitors

**DOI:** 10.1111/1759-7714.13594

**Published:** 2020-08-11

**Authors:** Yong Jun Choi, Taehee Kim, Eun Young Kim, Sang Hoon Lee, Do Sun Kwon, Yoon Soo Chang

**Affiliations:** ^1^ Department of Internal Medicine Yonsei University College of Medicine Seoul South Korea

**Keywords:** Hyperprogressive disease, NSCLC, PD‐L1 inhibitor, prognosis

## Abstract

**Background:**

Hyperprogressive disease (HPD) is a paradoxical acceleration of tumor growth after immune checkpoint inhibitor (ICI) treatment. This study aimed to identify the risk factors and to present a predictive model for HPD in patients treated with ICIs.

**Methods:**

A total of 78 non‐small cell lung cancer (NSCLC) cases, treated with at least two cycles of ICIs who underwent computed tomography (CT) for response assessment were recruited into the study from January 2016 to August 2019. HPD was defined by the following criteria: (i) time‐to‐treatment failure <2 months; (ii) a 50% increase in the sum of target lesion diameters; (iii) new development of at least two lesions in an already involved organ; (iv) appearance of a new organ lesion; and (v) a decrease in ECOG PS 2.

**Results:**

Of the 78 total patients, 15 (19.2%) had HPD. The risk factors of HPD were age; primary lesion size; and metastases in the contralateral lung, pleura, liver, and bone in multivariable logistic regression (odds ratio [OR]; 0.9038, 1.6619, 28.5913, 23.8264, 14.5711, and 20.1533, respectively, all *P*‐values < 0.05). By using these risk factors, we developed a prediction model for HPD and the area under the receiver operating characteristic curve of the model was 0.9556 (95% confidence interval [CI]: 0.9133–0.9978).

**Conclusions:**

HPD is relatively common and associated with a grave clinical outcome, requiring a careful monitoring in lung cancer patients treated with ICIs. Moreover, risk factors such as age, size of tumor and number of various metastatic lesions should be taken into consideration before ICI administration.

**Key points:**

**Significant findings of the study:**

Age, primary lesion size, and number of metastases are risk factors of HPD. HPD is strongly associated with poor prognosis. HPD during ICI use needs comprehensive monitoring.

**What this study adds:**

This is the first study to develop a prediction model. The area under the curve of the prediction model for HPD was 0.9556.

## Graphical abstract

HPD is strongly associated with poor prognosis. In this study, age, primary lesion size, and number of metastases were risk factors of HPD. The area under the curve of the prediction model for HPD was 0.9556.

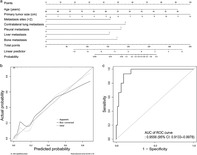

## Introduction

Lung cancer is one of the major causes of death, with 2.09 million newly diagnosed cases and 1.76 million mortality cases worldwide in 2018.^1^ Of the lung cancer cases, 85% are non‐small cell lung cancer (NSCLC), >50% show distant metastasis at the time of lung cancer diagnosis, and only 20% to 25% are identified as operable.^2^ For patients with nonoperable lung cancer, the treatment options include chemotherapy, targeted therapy, and immunotherapy.

In recent years, immunotherapy has emerged as an attractive treatment, especially monoclonal antibody‐based immune checkpoint inhibitor (ICI) therapy, which enhances the anticancer function of type T lymphocytes and increases the overall survival of patients with NSCLC.^3^, ^4^, ^5^ Since the Food and Drug Administration approval of nivolumab in March 2015 for metastatic squamous NSCLC to the recent approval of durvalumab in February 2018 for unresectable stage III NSCLC, the use of ICIs for NSCLC continues to expand.^6^, ^7^ However, like other cancer treatments, a variety of adverse events (AEs) have been reported with the use of ICIs. Particularly, immune‐related adverse events affecting the dermatologic, gastrointestinal, hepatic, endocrine, and other organ systems are issues of concern.^6^, ^8^

Recent studies reported a phenomenon known as hyperprogressive disease (HPD), in which a paradoxical acceleration of tumor growth occurs after ICI treatment. Unlike immune‐related adverse events, HPDs are known to occur relatively early. The prevalence of HPD in patients treated with ICIs has been reported to range from 7% to 29%, with differences depending on the study design.^9^, ^10^

HPD is a relatively common phenomenon. Therefore, clinicians should carefully review the possible adverse effects and the risk of HPD before initiating anti‐programmed death 1 (PD‐1)/programmed death ligand 1 (PD‐L1) therapy. However, there is no model or scoring system for predicting the development of HPD thus far.

This study aimed to identify the presence and risk factors of HPD after ICI therapy and to establish a model for predicting the development of HPD in Korean patients with NSCLC.

## Methods

The study was approved by the Institutional Review Board of Gangnam Severance Hospital (number: 3‐2019‐0229). The requirement for informed consent was waived due to the retrospective nature of this study.

### Study design and population

**Figure 1:**
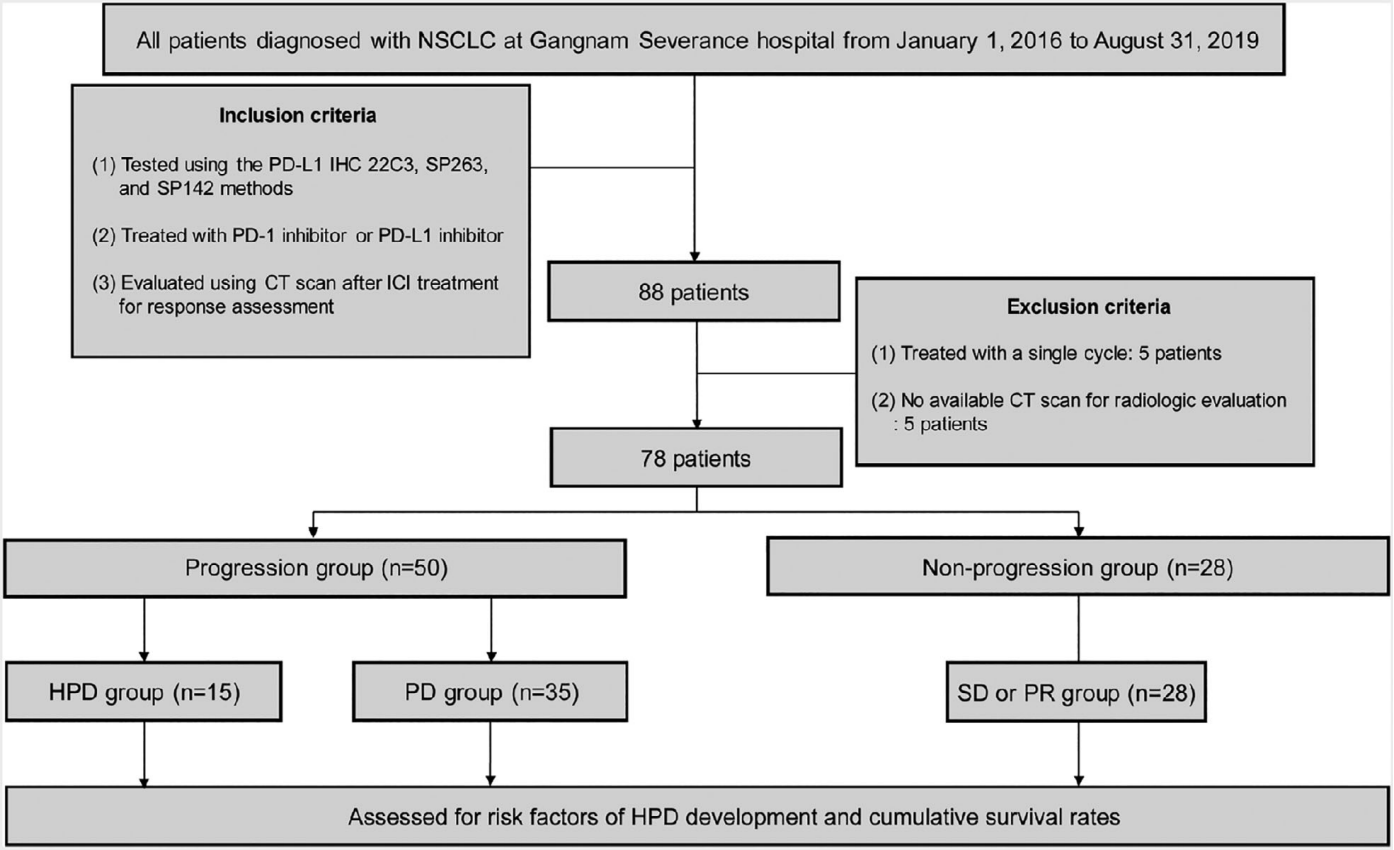
Diagram of study workflow.

This study was conducted through a review of electronic medical records at Gangnam Severance Hospital from 1 January 2016, to 31 August 2019. We included patients who were: (i) pathologically diagnosed with NSCLC; (ii) treated with at least two cycles of a PD‐1 inhibitor (nivolumab or pembrolizumab) or a PD‐L1 inhibitor (atezolizumab); and (iii) evaluated using computed tomography (CT) for response assessment after ICI treatment. We classified the enrolled patients into three groups according to the response to ICI treatment, and evaluated the risk factors of HPD (Fig 1). All data were collected in accordance with the amended Declaration of Helsinki. This study was approved by the institutional review board of Gangnam Severance Hospital (approval no. 3‐2019‐0229). Written informed consent was not required because of the retrospective study design.

### Data collection

We collected data on age, sex, smoking status, Eastern Cooperative Oncology Group performance scale (ECOG PS), neutrophil count, lymphocyte count, histologic type, genotype of mutation, type of ICI, line of ICI, date of ICI initiation, number of ICI administrations, previous anticancer treatment (history of chemotherapy, radiation therapy, targeted therapy, and steroid therapy), date of the most recent hospital visit, and status of death. Further, we assessed data on primary lesion size (maximum diameter measured on chest CT; in cases of recurrence, the primary lesion was defined as the largest mass), number of metastatic sites (count of involved solid organs, not all sites), status of specific metastasis (contralateral lung, pleura, nonregional lymph node, brain, liver, kidney, adrenal gland, and bone), and stage (according to the eighth edition of the tumor‐node‐metastasis [TNM] staging system). CT scan for radiologic evaluation was performed before PD‐1 or PD‐L1 inhibitor therapy and every eight weeks after treatment. According to the treatment response, the patients were classified as having progressive disease (PD), partial response (PR), and stable disease (SD) based on the first CT results after ICI treatment according to the Response Evaluation Criteria in Solid Tumors (RECIST) version 1.1. For survival analysis, overall survival was defined as the duration from the date of ICI initiation to the date of the most recent hospital visit. In addition, overall survival was compared by dividing all patients into three groups: HPD group, PD group, and nonprogressive (SD or PR) group.

### Definitions of HPD and pseudoprogression

HPD was defined using the following criteria in accordance with previous studies: (i) time‐to‐treatment failure <2 months (with treatment failure defined as ICI discontinuation because of cancer progression, drug toxicity, or death); (ii) a 50% increase in the sum of target lesion diameters between baseline and the first radiologic evaluation; (iii) new development of at least two lesions in an already involved organ between baseline and the first radiologic evaluation; (iv) appearance of a new organ lesion between baseline and the first radiologic evaluation; and (v) a decrease in ECOG PS 2 during the first two months of treatment.^11^ Patients who fulfilled at least three of the clinical/radiologic criteria were defined as exhibiting HPD, whereas those with RECIST 1.1 PD as the best response without fulfilling at least three criteria were defined as PD patients. All PR and SD patients were classified according to their RECIST 1.1 best response. Pseudoprogression (PP) was defined as progression at the first radiologic evaluation, which was redefined as PR or SD at reassessment at >6 months after ICI therapy.^12^

### Statistical analysis

To compare continuous variables, a parametric independent two‐sample *t*‐test or analysis of variance with Bonferroni's method was used. The Mann‐Whitney U‐test was used for nonparametric variables, based on the normality assumptions from the Shapiro‐Wilk test. The chi‐square test or Fisher's exact test was used to compare categorical variables. The risk factors of HPD development were analyzed using logistic regression, and the scoring system for predicting HPD development was expressed as a nomogram. In addition, Kaplan‐Meier analysis was used to compare the differences in survival rates among patient groups. Analysis was performed using R Statistical Package (version 3.6.2; Institute for Statistics and Mathematics, Vienna, Austria).

## Results

### Presentation of the HPD case

**Figure 2:**
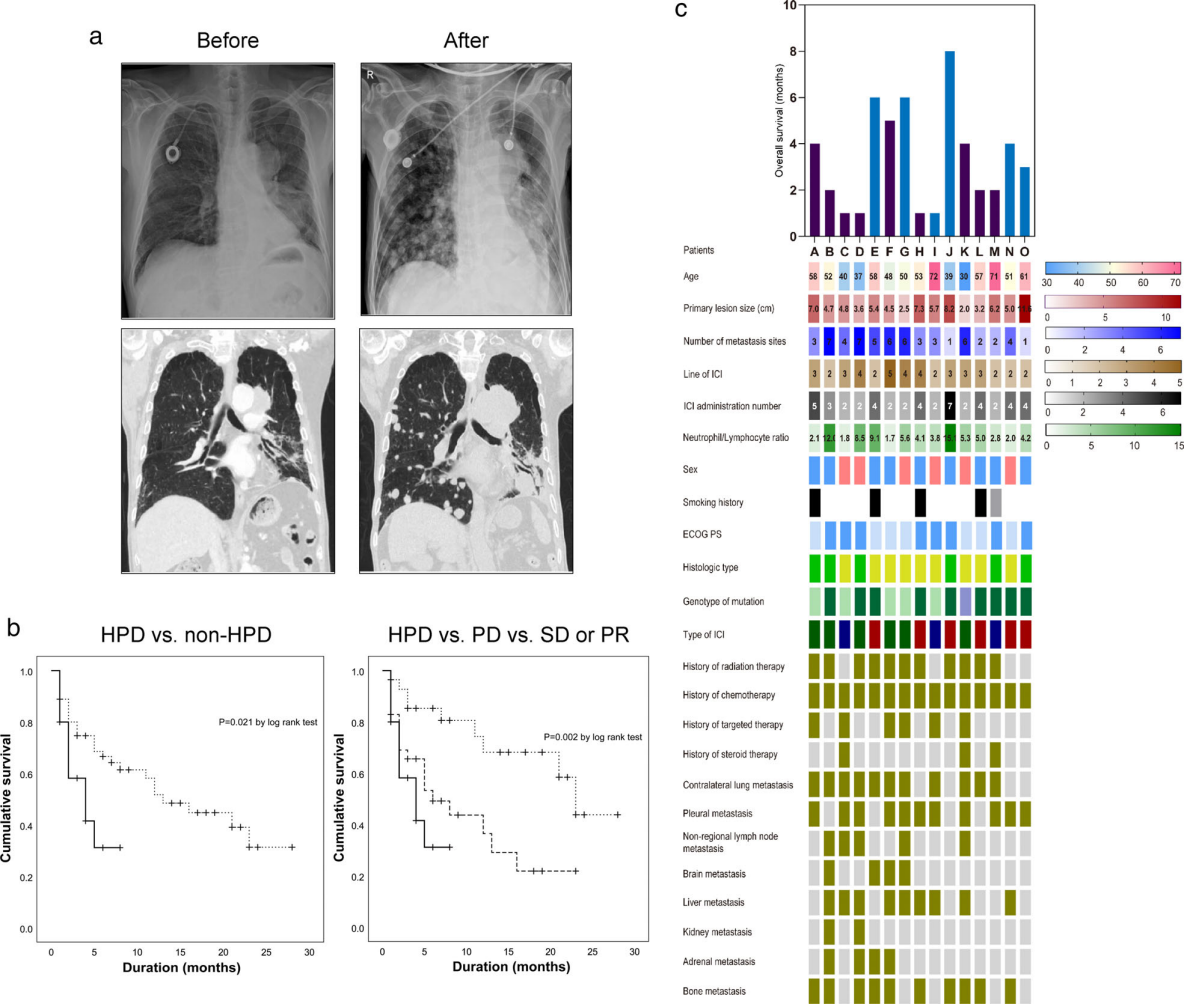
Characteristics of HPD patients. (**a**) Radiologic features of a HPD case before ICI treatment (left panel) and after two cycles of ICI treatment (right panel). (**b**) Kaplan‐Meier curve comparing overall survival between HPD and non‐HPD (left) and comparing overall survival among the HPD, PD, and SD or PD groups. Groups HPD (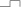), Non‐HPD (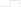); Groups HPD (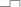), PD (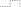), SD or PR (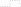) (**c**) Clinical features of HPD patients. (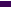) Dead, (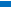) Alive. (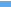) Male, (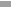) Female, (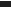) Ex‐smoker, (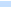) Current smoker, (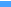) 0, (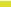) 1, (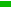) LUAD, (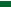) LUSC, (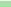) Wild type, (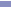) EGFR+, (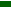) ALK+, (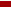) Nivolumab, (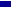) Pembrolizumab, (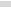) Atezolizumab, (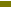) Positive, (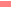) Negative. LUAD, lung adenocarcinoma; LUSC, lung squamous cell carcinoma.

A 71‐year‐old male patient with advanced pulmonary squamous cell carcinoma was admitted with progressive dyspnea and general weakness. He had been treated with concurrent chemoradiotherapy followed by navelbine‐carboplatin chemotherapy. Not long after planned treatment, he experienced disease progression and was then treated with the PD‐L1 inhibitor, atezolizumab. After two cycles of atezolizumab treatment, the patient complained of gastrointestinal symptoms (nausea, poor oral intake, and constipation), general weakness, dizziness, and worsening of dyspnea. Plain chest radiography showed multiple metastases in both lungs that had not been observed two months before and chest CT revealed extensive metastasis in both lungs, extending into the mediastinum, chest wall, and retroperitoneal space (Fig 2(a)). After confirmation of HPD, treatment with atezolizumab was discontinued but he suffered from progressive deterioration and died three weeks after diagnosis of HPD.

### Characteristics of the study cases

As in the case reported in this study, a rapid deterioration of clinical outcome is common in HPD patients. To estimate its incidence and identify risk factor of HPD, we recruited the lung cancer patients treated with ICIs from the study institute from January 2016, to August 2019. A total of 78 cases met the selection criteria and the clinical characteristics are presented in Table 1.

**Table 1:** Baseline characteristics of NSCLC patients treated with immune‐check point inhibitors (ICIs)

			Progression		Nonprogression	
Variables		Total	HPD	PD	SD or PR	*P*‐value
Number of patients (n [%])		78 (100)	15 (19.2)	35 (44.9)	28 (35.9)	
Continuous variables (mean ± standard deviation)						
Age, years		61.3 ± 11.3	51.8 ± 11.9	61.3 ± 8.9	66.4 ± 10.8	<0.001^**^
Primary lesion size, cm		4.3 ± 2.3	5.4 ± 2.4	4.4 ± 2.4	3.7 ± 1.7	0.045^*^
Number of metastatic sites		2.2 ± 1.7	4.0 ± 2.1	2.1 ± 1.5	1.3 ± 1.0	<0.001^**^
Line of ICI		2.8 ± 1.0	2.9 ± 1.0	2.8 ± 0.9	2.7 ± 1.0	0.708
ICI administration number		6.9 ± 6.3	3.5 ± 1.9	4.7 ± 2.9	11.3 ± 8.3	<0.001^**^
Neutrophils, 10^3^/μL		5.0 ± 3.4	5.2 ± 3.8	5.4 ± 4.1	4.5 ± 2.0	0.499
Lymphocytes, 10^3^/μL		1.3 ± 0.7	1.2 ± 0.7	1.1 ± 0.7	1.5 ± 0.7	0.158
Neutrophil/lymphocyte ratio		5.7 ± 6.9	5.5 ± 4.0	7.0 ± 9.3	4.2 ± 3.6	0.274
Categorical variables (n [%])						
Sex	Male	49 (62.8)	9 (60.0)	19 (54.3)	21 (75.0)	0.245
	Female	29 (37.2)	6 (40.0)	16 (45.7)	7 (25.0)	
Smoking history	Never smoker	43 (55.1)	10 (66.7)	19 (54.3)	14 (50.0)	^†^0.536
	Ex‐smoker	8 (10.3)	1 (6.6)	2 (5.7)	5 (17.9)	
	Current smoker	27 (34.6)	4 (26.7)	14 (40.0)	9 (32.1)	
ECOG PS	0	33 (42.3)	7 (46.7)	14 (40.0)	12 (42.9)	^†^0.980
	1	44 (56.4)	8 (53.3)	20 (57.1)	16 (57.1)	
	2	1 (1.3)	0 (0)	1 (2.9)	0 (0)	
Histologic type						
Adenocarcinoma		47 (60.3)	9 (60.0)	23 (65.7)	15 (53.6)	^†^0.715
Squamous cell carcinoma		30 (38.5)	6 (40.0)	12 (34.3)	12 (42.9)	
Adenosquamous cell carcinoma		1 (1.2)	0 (0.0)	0 (0.0)	1 (3.5)	
Stage	III	8 (10.3)	0 (0)	4 (11.4)	4 (14.3)	^†^0.439
	IV	70 (89.7)	15 (100)	31 (88.6)	24 (85.7)	
T stage	T1	5 (6.4)	0 (0)	2 (5.6)	3 (10.7)	^†^0.033^*^
	T2	15 (19.2)	0 (0)	8 (22.9)	7 (25.0)	
	T3	8 (10.3)	0 (0)	3 (8.6)	5 (17.9)	
	T4	50 (64.1)	15 (100)	22 (62.9)	13 (46.4)	
Genotype of mutation	Wild‐type	55 (70.5)	9 (60.0)	24 (68.6)	22 (78.6)	^†^0.763
	EGFR	19 (24.4)	5 (33.3)	9 (25.6)	5 (17.9)	
	ALK	3 (3.8)	1 (6.7)	1 (2.9)	1 (3.5)	
	ROS	1 (1.3)	0 (0.0)	1 (2.9)	0 (0.0)	
Type of ICI	Nivolumab	30 (38.5)	6 (40.0)	13 (37.1)	11 (39.3)	^†^0.729
	Pembrolizumab	40 (51.3)	6 (40.0)	19 (54.3)	15 (53.6)	
	Atezolizumab	8 (10.2)	3 (20.0)	3 (8.6)	2 (7.1)	
Previous anticancer treatment						
History of radiation therapy		45 (57.7)	11 (73.3)	21 (60.0)	13 (46.4)	0.234
History of chemotherapy		74 (94.9)	15 (100.0)	34 (97.1)	25 (89.3)	^†^0.400
History of targeted therapy		22 (28.2)	6 (40.0)	9 (25.7)	7 (25.0)	0.608
History of steroid therapy		18 (23.1)	3 (20.0)	7 (20.0)	8 (28.6)	0.731
Metastatic sites						
Contralateral lung		31 (39.7)	11 (73.3)	12 (34.3)	8 (28.6)	0.011^*^
Pleura		37 (47.4)	11 (73.3)	15 (42.9)	11 (39.3)	0.084
Nonregional lymph node		16 (20.5)	5 (33.3)	5 (14.3)	6 (21.4)	0.334
Brain		22 (28.2)	4 (26.7)	12(34.3)	6 (21.4)	0.547
Liver		15 (19.2)	9 (60.0)	6 (17.1)	0 (0.0)	<0.001^**^
Kidney		4 (5.1)	2 (13.3)	2 (5.7)	0 (0.0)	^†^0.178
Adrenal gland		11 (14.1)	4 (26.7)	6 (17.1)	1 (3.6)	^†^0.065
Bone		27 (34.6)	10 (66.7)	13 (37.1)	4 (14.3)	0.002^*^

ALK, anaplastic lymphoma receptor tyrosine kinase; ECOG PS, Eastern Cooperative Oncology Group performance status; EGFR, epidermal growth factor receptor; HPD, hyperprogressive disease; ICI, immune‐check point inhibitor; NSCLC, non‐small cell lung carcinoma; PD, progressive disease; PR, partial response; SD, stable disease.

* *P* < 0.05.

** *P* < 0.001.

^†^ Fisher exact test.

The mean age of all enrolled patients was 61.3 ± 11.3 years, of whom 49 (62.8%) were men and 43 (55.1%) were never‐smokers. According to histologic type, 47 (60.3%) cases were adenocarcinoma and 30 (38.5%) cases were squamous cell carcinoma. The pathologic stage was stage III in eight patients (10.3%) and stage IV in 70 patients (89.7%); thus, most of the enrolled patients had advanced lung cancer. The type of ICI used was nivolumab in 30 patients (38.5%), pembrolizumab in 40 patients (51.3%), and atezolizumab in eight patients (10.2%). The treatment response after immunotherapy was as follows: HPD (15 cases, 19.2%), PD (35 cases, 44.9%), and SD or PR (28 cases, 35.9%). PP was observed in four cases (4.9%), which were classified into either the SD or PR during follow‐up. The median overall survival for all patients was 12 months.

The effect of HPD development after ICI treatment on prognosis was then evaluated in these patient groups. In the comparison between the HPD and non‐HPD groups, the median overall survival was four and 13 months (*P* = 0.021 by log‐rank test, Fig 2(b), left panel). When the cases were further classified into the HPD, PD, and SD or PR groups and analyzed, the median survival of each group was four, six, and 23 months (*P* = 0.002 by log‐rank test, Fig 2(b), right panel). These findings indicate that the NSCLC patients who experienced HPD had significant poor clinical outcome, requiring risk factor identification and prediction model to prevent HPD.

### Risk factor of HPD

**Figure 3:**
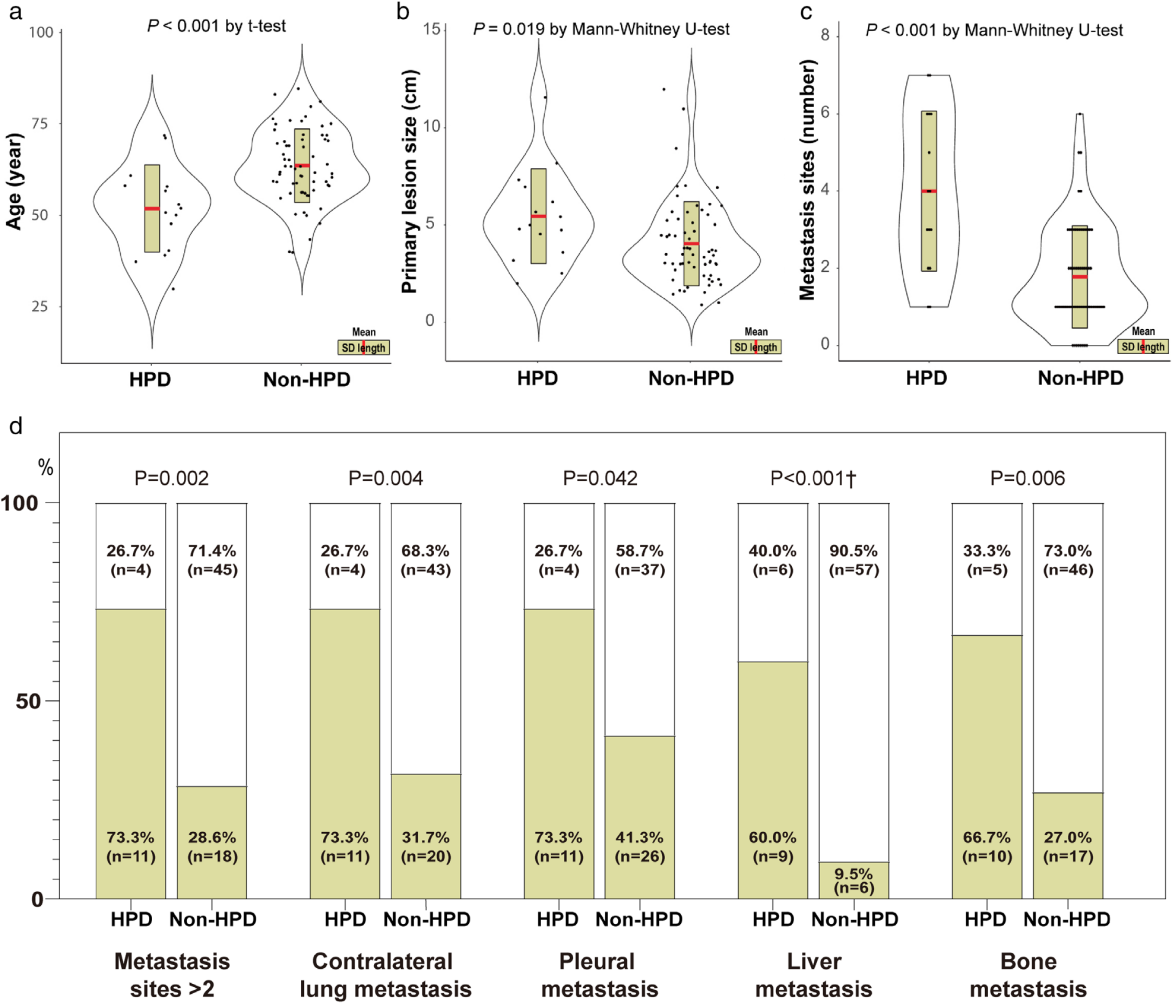
Comparison between the HPD and non‐HPD groups according to (**a**) the age; (**b**) primary tumor size; (**c**) total number of metastatic sites; and (**d**) characteristics of the metastasis. (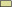) Positive, (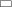) Negative.

To predict the risk factors of HPD that may occur after ICI treatment, and to devise countermeasures, further investigation was conducted on the NSCLC cases who were treated with ICIs. The clinical features of 15 HPD patients are shown in Fig 2(c). Next, the characteristics of HPD patients and non‐HPD patients were compared. The mean age of HPD patients was younger than that of non‐HPD patients (51.8 ± 11.9 vs. 63.5 ± 10.0 years, *P* < 0.001 by *t*‐test) (Fig 3(a)). In addition, HPD patients had higher mean values of primary lesion size and number of metastatic sites than non‐HPD patients (5.4 ± 2.4 vs. 4.0 ± 2.2 cm, *P* = 0.019 by Mann‐Whitney U‐test; 4.0 ± 2.1 vs. 1.78 ± 1.3, *P* < 0.001 by Mann‐Whitney U‐test, respectively) (Fig 3(b,c)). With respect to the metastatic sites at baseline, the HPD patient group had significantly more patients with more than two metastatic sites, and the number of patients with contralateral lung, pleura, liver, and bone metastases also significantly increased (Fig 3(d)).

### Modeling of HPD

To predict the development of HPD, we conducted univariable logistic regression using parameters including age, primary lesion size, number of metastatic sites, line of ICI, number of ICI administrations, neutrophil count, lymphocyte count, neutrophil/lymphocyte ratio, sex, smoking history, ECOG PS, histologic type, TNM stage, T stage, genotype of mutation, type of ICI, previous anticancer treatment, and presence of distant metastasis (contralateral lung, pleura, nonregional lymph node, brain, liver, kidney, adrenal gland, and bone). In univariable logistic regression for HPD development, younger age, larger primary lesion size, and greater number of metastatic sites were associated with a significantly higher tendency for HPD development (odds ratio [OR] 0.9007 [95% CI: 0.8389–0.9548], *P* = 0.001; 1.2783 [1.0127–1.6427], *P* = 0.041; 2.1291 [1.4827–3.3317], *P* < 0.001, respectively) (Appendix S1). In particular, the presence of metastasis in the contralateral lung, pleura, liver, and bone significantly correlated with HPD development (OR 5.9125 [95% CI: 1.784–23.4944], *P* = 0.006; 3.9135 [1.1937–15.3789], *P* = 0.032; 14.2500 [3.9275–58.2402], *P* < 0.001; 5.4118 [1.6768–19.5916], *P* = 0.006, respectively).

Multivariable logistic regression analysis for HPD prediction was then performed using variables with *P* < 0.05. In multivariable analysis, age, primary lesion size, and presence of metastasis (in the contralateral lung, pleura, liver, and bone) were significantly correlated with HPD (OR 0.9038 [95% CI: 0.8071–0.9847], *P* = 0.038; 1.6619 [1.1554–2.7076], *P* = 0.014; 28.5913 [2.4931–1108.5334], *P* = 0.023; 23.8264 [2.2913–644.7076], *P* = 0.023; 14.5711 [1.7200–207.7863], *P* = 0.023; and 20.1533 [2.1647–396.6042], *P* = 0.019, respectively) (Table 2).

**Table 2:** Multivariable logistic regression of predicting models for HPD development

Variables	OR (95% CI)	Coefficients	AUC of ROC	*P*‐value	VIF	Hosmer‐Lemeshow test
Age, years	0.9038 (0.8071–0.9847)	−0.101 19	0.9556 (0.9133–0.9978)	0.038	1.339 93	*P* = 0.998 274 6
Primary lesion size, cm	1.6619 (1.1554–2.7076)	0.507 99		0.014	1.06204	
Number of metastatic sites >2	0.0660 (0.0021–1.0134)	−2.718 78		0.075	2.219 85	
Contralateral lung metastasis	28.5913 (2.4931–1108.5334)	3.353 10		0.023	1.401 37	
Pleural metastasis	23.8264 (2.2913–644.7076)	3.170 80		0.023	1.120 03	
Liver metastasis	14.5711 (1.7200–207.7863)	2.679 04		0.023	1.534 04	
Bone metastasis	20.1533 (2.1647–396.6042)	3.170 80		0.019	1.331 66	

AUC, area under the curve; ROC, receiver operating characteristic curve; VIF, variance inflation factor.

**Figure 4:**
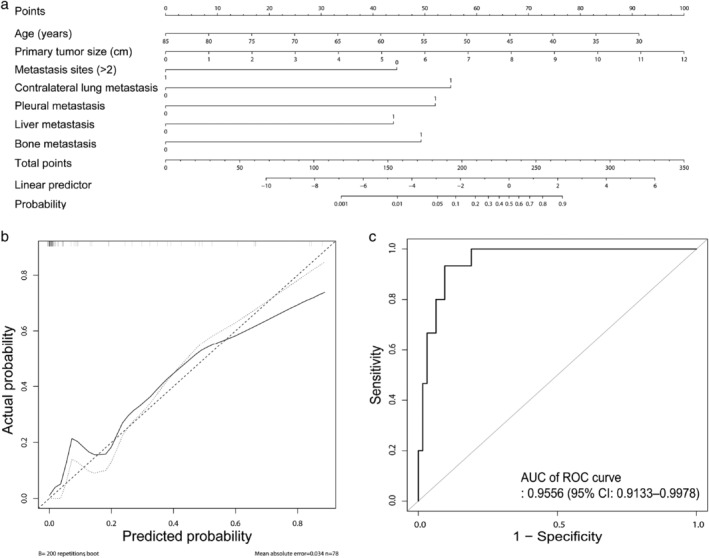
Prediction model for development of HPD. (**a**) Nomogram for the probability of HPD development. (**b**) Calibration plot, Apparent: (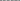) Bias‐corrected, (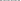) Ideal, (**c**) Receiver operating characteristic curves of the prediction model. (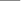) ROC curve.

On the basis of these results, we established a model for predicting HPD using variables including age, primary lesion size, and presence of metastasis (in the contralateral lung, pleura, liver, and bone). To examine the correlations between variables, a correlation plot was drawn (Appendix S2). Among the variables, smoking status was significantly associated with male sex. Moreover, age was negatively correlated with the number of metastatic sites and the presence of liver or bone metastasis. Multicollinearity tests were performed to confirm the interaction between these variables. The variance inflation factor scores for each variable were all <10, with no interaction between the variables (Table 2). To test the suitability of the model, the Hosmer‐Lemeshow test was conducted and the *P*‐value was found to be >0.05 (*P* = 0.998 274 6). A nomogram for the prediction model is described in Fig 4(a). The actual and predicted values showed good agreement, confirming that the model is suitable (Fig 4(b)). The predicted probability of the model is described in Fig 4(c). Area under the receiver operating characteristic curve (AUC) was 0.9556 (95% CI: 0.9133–0.9978).

## Discussion

Until recently, there has been no established definition of HPD. For this reason, several studies have proposed objective and easy‐to‐measure criteria. The present study defined HPD based on the RECIST 1.1 criteria; however, because RECIST is a one‐dimensional tumor assessment, a recent study suggested that a more accurate evaluation is possible by using volumetric measurement.^13^

We found that age was significantly related to the development of HPD. The results of the correlation between age and HPD prevalence in previous studies are controversial. Ferrara *et al*. and Kanjanapan *et al*. did not find any significant association between HPD and age.^12^, ^14^ In addition, a recent meta‐analysis reported that age > 65 years was not correlated with the development of HPD.^15^ However, Champiat *et al*. reported that patients with HPD were older than those without HPD.^16^ The difference in results among studies is attributed to the small number of HPD patients, as well as to variations in the patient group and the definition of HPD. In this study, the age of the enrolled patients was negatively correlated with the number of metastatic sites, presence of liver metastasis, and presence of bone metastasis. As a result, age may be related to HPD; however, studies with larger populations and involving multiple center studies are needed.

The primary lesion size and number of metastatic sites were positively correlated with HPD development in this study. Therefore, it is possible that the tumor burden is associated with the development of HPD. Although TNM stage or T stage was not significantly related to HPD development in this study, this was attributed to the advanced disease stages (stage IV and T4) of all HPD patients enrolled in this study. Conversely, several studies have reported no significant association between other markers of tumor burden (sum of the largest diameter of target lesions at baseline, tumor volume at baseline on volumetric measurement) and HPD development.^13^, ^17^, ^18^ However, these studies had different definitions of HPD and inclusion criteria. Therefore, multicenter studies with a consistent design are warranted.

Presence of metastasis in the contralateral lung and pleura also showed a positive correlation with HPD development in this study. There was no obvious evidence for association between contralateral lung and pleural metastasis, and HPD. However, in some previous studies, pleural and contralateral lung metastasis was correlated with poor prognosis in NSCLC patients treated with ICIs.^19^, ^20^, ^21^, ^22^, ^23^ Because contralateral lung and pleural metastasis may reflect tumor burden or spread, in the aspect of tumor burden, pleural and contralateral metastasis can affect the prognosis in NSCLC patients treated with ICIs.^24^ Huang *et al*. reported that poor clinical outcome was the result of an imbalance between T cell reinvigoration and tumor burden.^25^ The magnitude of reinvigoration of circulating exhausted‐phenotype CD8 T cells determined in relation to the baseline tumor burden correlated with clinical response. Therefore, if there is a greater baseline tumor burden this may be associated with a worse prognosis. However, the association with HPD is still unclear, and further in depth studies are required.

Liver and bone metastases were also found to be highly related to the development of HPD. Many previous studies have reported a correlation between liver metastasis and poor prognosis after ICI therapy or hyperprogression; however, the exact mechanism remains unclear.^11^, ^26^, ^27^, ^28^, ^29^, ^30^ Several hypotheses have been proposed to explain the mechanism. Tumeh *et al*.^30^ reported that melanoma patients with liver metastases had a reduced density of CD8+ T cells at the invasive tumor margin in distant tumors compared with patients without liver metastases, which is associated with the response to a PD‐1 inhibitor. Lee *et al*.^31^ reported that patients with liver metastasis had significantly lower CD8/Foxp3^+^ regulatory T cell ratio and decreased percentage of activated PD‐1^+^/CTLA‐4^+^ CD8 cells from biopsy samples. These results suggest liver‐induced peripheral and systemic immune tolerance, which lead to poor responses to PD‐1/PD‐L1 inhibitor therapy and can increase the probability of HPD development in patients with liver metastases.^31^ In addition, patients with liver metastases may also have other baseline characteristics associated with HPD, such as multiple metastatic sites, resulting in a higher risk for HPD.^15^, ^24^ Although there are few studies on the direct relationship between bone metastasis and HPD development, bone metastasis has been reported to be a risk factor associated with the prognosis after immunotherapy.^32^, ^33^ Because the bone marrow serves as a substitute for secondary lymphoid tissue through the primary immune response or memory response, bone metastasis can affect the modulation of the immune‐response role of the bone.^32^, ^34^, ^35^ Conversely, because bone metastasis is also significantly accompanied by liver metastasis, bone metastasis may also show an association with HPD.^32^ However, because studies on the relationship between HPD and bone metastasis are insufficient, large‐scale multicenter studies are warranted.

The metastatic site is associated not only with HPD but also with the treatment responsiveness and prognosis after ICI treatment.^24^, ^36^, ^37^ As metastasis results in decreased function of the involved organs in addition to the tumor burden, a comprehensive understanding of various metastatic sites is necessary prior to immune therapy.

Although this study did not broadly include blood markers, the levels of lactate dehydrogenase (LDH) may be related to HPD in several studies. Kim *et al*. reported LDH levels above the upper normal limit were significantly related with HPD.^26^ On the contrary, in a study by Sadal *et al*. LDH showed no meaningful results.^17^ In a recent meta‐analysis, LDH above the upper normal limit was significantly correlated with HPD.^15^ Furthermore, Sasaki *et al*. reported that absolute neutrophil count, neutrophil‐to‐lymphocyte ratio, and C‐reactive protein above the median value were also associated with HPD development.^15^, ^28^ Consequently, these blood biomarkers reflect tissue damage and inflammatory reactions. Further studies are needed on biomarkers that can predict drug response.

A strength of this study is that it investigated the risk factors of HPD that have not been previously established. Moreover, this is also the first study to develop a prediction model. However, this study also had some limitations. First, the number of enrolled patients was small, which hinders the factors that could actually affect HPD from reaching statistical significance. This is a common limitation in other previous studies, and multicenter studies are needed to overcome this problem. Second, the tumor burden is expected to be important for HPD diagnosis and prognosis, and in this study it was not evaluated using volumetric measurement, which may have affected the results. Third, the patient groups in this study were sampled and analyzed through a retrospective review of electronic medical records, which can cause biases in the process and affect the results. Fourth, the HPD prediction model presented in this study has not been externally validated in other patient groups. A more accurate assessment of this model would require its verification in new patient groups.

In conclusion, HPD is relatively common in patients treated with ICIs and is associated with a poor prognosis. Therefore, it should be carefully monitored in patients with NSCLC who are receiving ICI therapy and the risk factors of HPD need to be evaluated before ICI therapy.

## Disclosure

The authors report that there are no conflicts of interest.

## Supporting information


**Appendix S1.** Univariable logistic regression for HPD development

**Appendix S2.** Correlation plot among the parameter of baseline characteristics
