# Supplementary material for: Prediction model for hyperprogressive disease in non‐small cell lung cancer treated with immune checkpoint inhibitors
Source: Thorac Cancer. 2020 Aug 11;11(10):2793–803. doi: 10.1111/1759-7714.13594 (PMC7529559; doi:10.1111/1759-7714.13594)
Supplement: Supplementary file 1 — Appendix S1. Univariable logistic regression for HPD development [file TCA-11-2793-s001.docx]

| **Supplementary Data 1. Univariable logistic regression for HPD development** | | | |
| --- | --- | --- | --- |
| **Variables** | **OR (95% CI)** | **Coefficients** | **P-value** |
| **Continous variables** |  |  |  |
| Age, years | 0.9007(0.8389-0.9548) | -0.1046 | 0.001^*^ |
| Primary lesion size, cm | 1.2783(1.0127-1.6427) | 0.2455 | 0.041^*^ |
| Number of metastasis sites | 2.1291(1.4827-3.3317) | 0.7557 | <0.001^**^ |
| Line of ICI | 1.2225(0.6769-2.1928) | 0.2009 | 0.497 |
| ICI administration number | 0.8724(0.7167-0.9950) | -0.1366 | 0.098 |
| Neutrophils, 10^3/μL | 1.0160(0.8420-1.1808) | 0.0158 | 0.846 |
| Lymphocytes, 10^3/μL | 0.7609(0.3138-1.7048) | -0.2732 | 0.521 |
| Neutrophil/Lymphocyte ratio | 0.9955(0.8867-1.0726) | -0.0045 | 0.918 |
| **Categorical variables** |  |  |  |
| Sex |  |  |  |
| Male | 1.0000 |  |  |
| Female | 1.1594(0.3496-3.6388) | 0.1479 | 0.802 |
| Smoking history |  |  |  |
| Never smoker | 1.0000 |  |  |
| Ever-smoker | 0.5500(0.1563-1.7350) | -0.5978 | 0.321 |
| Current smoker | 0.6324(0.1607-2.0916) | -0.4582 | 0.474 |
| ECOG PS | 0.7704(0.2537-2.3056) | -0.2609 | 0.639 |
| Histologic type |  |  |  |
| Adenocarcinoma | 0.9868(0.3158-3.2634) | -0.0133 | 0.982 |
| Squamous cell carcinoma | 1.0833(0.3272-3.3920) | 0.0800 | 0.892 |
| Adenosquamous cell carcinoma | 0.0000 (NA) | -14.0997 | 0.992 |
| Stage |  |  |  |
| III | 1.0000 |  |  |
| IV | 11603131(0-NA) | 16.2700 | 0.991 |
| T stage | 37052024(0-NA) | 17.4300 | 0.993 |
| Genotype of mutation |  |  |  |
| Wild type | 1.0000 |  |  |
| EGFR | 1.7500(0.4806-5.8363) | 0.5596 | 0.371 |
| ALK | 2.1786(0.0972-24.3426) | 0.7787 | 0.537 |
| ROS | 0.0000 (NA) | -14.1470 | 0.992 |
| Type of ICI |  |  |  |
| Nivolumab | 1.0833(0.3272-3.3920) | 0.0800 | 0.892 |
| Pembrolizumab | 0.5333(0.1614-1.6565) | -0.6286 | 0.282 |
| Atezolizumab | 3.6875(0.6579-18.9279) | 1.3049 | 0.115 |
| Previous anti-cancer treatment |  |  |  |
| History of radiation therapy | 2.3456(0.7160-9.1884) | 0.8525 | 0.180 |
| History of chemotherapy | 10816478(0.0000-NA) | 16.2000 | 0.994 |
| History of targeted therapy | 1.9583(0.5788-6.3313) | 0.6721 | 0.264 |
| History of steroid therapy | 0.8000(0.1664-2.9381) | -0.2231 | 0.753 |
| Metastasis sites |  |  |  |
| Number of metasis sites > 2 | 6.8750(2.0610-27.4939) | 1.9279 | 0.003^*^ |
| Contralateral lung | 5.9125(1.784-23.4944) | 1.7771 | 0.006^*^ |
| Pleura | 3.9135(1.1937-15.3789) | 1.3644 | 0.032^*^ |
| Non-regional lymph node | 2.3636(0.6351-8.1667) | 0.8602 | 0.179 |
| Brain | 0.9091(0.2286-3.0611) | -0.0953 | 0.883 |
| Liver | 14.2500(3.9275-58.2402) | 2.6568 | < 0.001^**^ |
| Kidney | 4.6923(0.5247-42.1548) | 1.5459 | 0.139 |
| Adrenal gland | 2.9091(0.6704-11.4794) | 1.0678 | 0.132 |
| Bone | 5.4118(1.6768-19.5916) | 1.6886 | 0.006^*^ |

HPD, hyperprogressive disease; ICI, immune-check point inhibitor; ECOG PS, Eastern Cooperative Oncology Group performance status; EGFR, epidermal growth factor receptor; ALK, anaplastic lymphoma receptor tyrosine kinase; * P<0.05, ** P<0.001
