# Supplementary figures and images for: Prediction model for hyperprogressive disease in non‐small cell lung cancer treated with immune checkpoint inhibitors
Source: Thorac Cancer. 2020 Aug 11;11(10):2793–803. doi: 10.1111/1759-7714.13594 (PMC7529559; doi:10.1111/1759-7714.13594)

## Slide 1
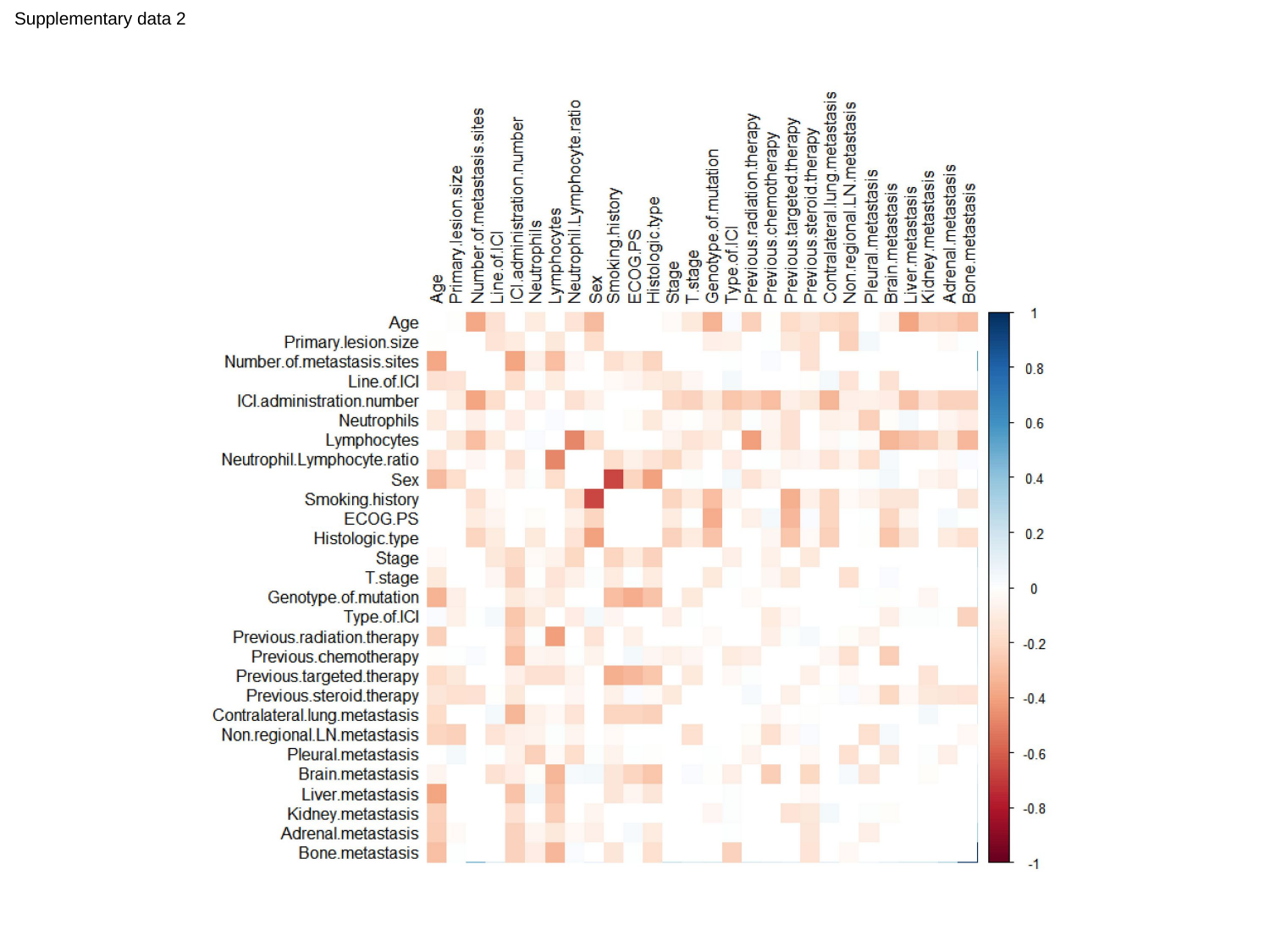

Supplementary data 2

Supplement: Supplementary file 2 — Appendix S2. Correlation plot among the parameter of baseline characteristics [file TCA-11-2793-s002.pptx]
